# Effect of chemotherapy on gut microbiota in patients with colorectal cancer

**DOI:** 10.3389/fmicb.2026.1811645

**Published:** 2026-07-13

**Authors:** Xiaoying Mi, Peng Zheng, Shiyu Zhao, Xiaoping Zhang, Lu Tian, Xiangbai Wu

**Affiliations:** 1 Department of Colorectal and Anal Surgery, The Second People's Hospital of China Three Gorges University, Yichang, Hubei, China; 2 Hubei Clinical Medical Research Center for Precision Prevention and Treatment of Gastro Gut Cancer in Elderly People, Yichang, Hubei, China

**Keywords:** 16srRNA, chemotherapy, chemotherapy side effects, colorectal cancer, gut microbiota

## Abstract

**Introduction:**

The gut microbiota plays a crucial role in digestion, energy conversion, immune regulation, and disease resistance. Research has established a link between gut microbiota and the development and progression of colorectal cancer(CRC), noting variations in their composition within and around tumors, and gut microbiota may impact the effectiveness of drug therapies. Chemotherapy remains the cornerstone of CRC treatment, yet studies exploring its relationship with gut microbiota are still limited.

**Methods:**

In this study, 16S rRNA sequencing was performed to profile gut microbiota alterations in 20 patients with colorectal adenocarcinoma before and after chemotherapy, and to further explore the association between first-line chemotherapy and gut microbiota composition. All participants received the FOLFOX6 chemotherapy regimen, and fecal samples were collected pre- and post-treatment, aiming to elucidate the connection between chemotherapy and gut microbiota by analyzing alterations in microbial composition, diversity, and richness across different chemotherapy cycles.

**Results:**

Following the first chemotherapy cycle, Chao1 index indacate there is a decline in gut microbiota richness among CRC patients (P < 0.01), along with notable shifts in microbial composition. Specifically, the phyla Firmicutes and Bacteroides decreased, while Proteobacteria, Actinobacteria, and Verrucomicrobia increased in abundance. Comparative analyses of gut microbiota profiles across sequential chemotherapy cycles revealed no statistically significant intergroup differences in the Chao 1 and Simpson indices (all P > 0.05). As chemotherapy cycles proceeded, the gut microflora did not show regular dynamic changes with the progression of chemotherapy cycles. KEGG functional gene prediction analysis indicated that chemotherapy-induced alterations in gut microbiota composition were linked to common chemotherapy side effects, including metabolic, immune, hematological, and neurological disturbances.

**Discussion:**

Our results demonstrate that chemotherapy has a certain correlation with the composition of gut microbiota in patients with colorectal cancer, and these microbial changes show a potential association with chemotherapy-related adverse events.

## 1 Introduction

Colorectal cancer (CRC) ranks as the third most prevalent malignant tumor globally, with consistently high incidence and mortality rates (Eng et al., 2024). In 2020 alone, there were an estimated 1.93 million new CRC cases worldwide, accounting for roughly 10% of all cancer diagnoses, and resulting in 935,173 deaths, making it the second leading cause of cancer-related mortality globally (Sung et al., 2021). Projections from GLOBOCAN 2020 suggest that the number of new CRC cases will surge from 1,931,590 in 2020 to 3,154,674 by 2040, representing a 63.3% increase (Siegel et al., 2020). This trend underscores the growing global burden of CRC, highlighting the urgent need for enhanced attention and effective prevention and intervention strategies. Oxaliplatin-based chemotherapy is the standard treatment for CRC, but the prognosis for CRC patients remains poor due to recurrence and metastasis, often stemming from chemotherapy intolerance, such as severe gastrointestinal reactions and bone marrow suppression (Yu et al., 2021). Recent research has revealed that the gut microbiota plays a pivotal role in various physiological processes, including metabolism, immune regulation, and inflammatory responses, and is closely linked to human health and disease progression, including CRC (Gadaleta et al., 2020; Mahnaz et al., 2021; Shah and Itzkowitz, 2022; Zhao et al., 2019). Dysbiosis of the gut microbiota, such as the abnormal abundance of *Fusobacterium nucleatum and pks*+ *E.coli* mutations, has been found in CRC patients and may elevate cancer risk by promoting inflammation and genetic instability (Chen et al., 2023; Ou et al., 2022). Moreover, studies have demonstrated that the gut microbiota can influence CRC treatment. Modulating the interaction between gut microbiota and anti-tumor immunity can enhance the efficacy of CRC immunotherapy. Li et al. (2021) research also suggests that variations in *Roseburia faeci* abundance may serve as a potential biomarker for predicting chemotherapy efficacy, offering new insights into personalized CRC treatment (Zhuang et al., 2023). However, research on the relationship between gut microbiota and CRC chemotherapy remains limited, and most studies have focused solely on comparing gut microbiota differences between CRC patients and healthy individuals, which fails to fully account for individual variations.

In this study, 16S rRNA gene sequencing was used to analyze dynamic shifts in gut microbiota structure of colorectal cancer patients before chemotherapy and across successive treatment cycles. We also preliminarily explored the potential association between intestinal microbes and common chemotherapy-related adverse reactions.

## 2 Materials and methods

### 2.1 Patient inclusion criteria and ethical approval

Patients who underwent radical surgery for CRC at the Second People's Hospital of China Three Gorges University between December 2022 and November 2023 were screened. Only those with a pathological diagnosis of colorectal adenocarcinoma, as per predefined inclusion and exclusion criteria, were considered. They were subsequently recruited from three predefined dietary pattern groups: balanced meat-vegetable intake, high meat consumption, and vegetable-predominant diet, with comparable sample sizes ensured across groups to minimize potential bias from dietary patterns on gut microbiota composition. Ultimately, a total of 20 patients aged 50–80 years were recruited. Baseline characteristics including gender, age, nutritional status and daily living habits of the enrolled patients are shown in Table 1. To minimize age-related confounding, this study included patients >50 years old with late-onset colorectal cancer, while excluding those >90 years old, as gut microbiota composition varies with age and shows functional decline in nonagenarians (Hopkins et al., 2002; Xu et al., 2019). All patients in this study followed standard clinical protocols. The standardized FOLFOX6 regimen (oxaliplatin plus calcium folinate plus 5-fluorouracil) was administered every 2 weeks. To avoid confounding effects from variations in postoperative recovery time, all patients initiated chemotherapy at week 4 after surgery. No participants had taken antibiotics in the 3 months before enrollment. All patients received short-course prophylactic cephalosporins from 24 h preoperatively to 48 h postoperatively, which were completely withdrawn for the remaining perioperative period until chemotherapy commenced. Short-term antiemetics were prescribed only for patients presenting with mild gastrointestinal adverse events during chemotherapy. Antibiotics, proton pump inhibitors, and exogenous probiotics were prohibited throughout the entire study, so as to eliminate confounding effects on intestinal microbiota testing (Table 2). Based on the research design and observation timeline, participants were divided into four groups: baseline (pre-chemotherapy), the first group (post-cycle 1), the mid-term group (post-cycle 3) and the endpoint group (post-cycle 6). Considering clinical practice and published evidence that adverse reactions to chemotherapy mostly emerge in cycles 3–4, an additional sampling time point after cycle 4 was included in this study. Fecal specimens were collected 24 h prior to the first chemotherapy administration and 24 h after the end of cycles 1, 3, 4, and 6.

**Table 1:** Baseline data of enrolled patients.

Serial number	Gender	Age	BMI	Preoperative serum albumin levels g/L	Level of nutrition	Whether antibiotics were taken before sampling	Diet 0. Even meat and vegetables 1. A lot of meat 2. Vegetables predominate	Weekly defecation situation 0.4–7 times 1. ≤ 3 times 2. ≥8 times
RAG34810	Female	57	21.76	42.7	Normal	No	2	0
S01669032	Female	79	29.55	47.1	Obese	No	0	0
S0187673746	Female	57	21.76	45.3	Normal	No	2	0
S0187686289	Female	50	21.23	43.3	Normal	No	2	0
S0187692156	Male	61	29.34	48.6	Normal	No	1	0
S0187693323	Female	51	23.44	47.9	Normal	No	2	0
S0187693536	Male	67	23.18	40.2	Normal	No	1	0
S0187698716	Female	70	22.66	44.2	Normal	No	1	0
S0187698873	Male	77	21.26	44	Normal	No	1	0
S0187698985	Female	50	21.23	49.5	Normal	No	2	0
S0187699051	Female	74	27.18	43.8	Overweight	No	2	0
S0187701736	Female	75	26.56	39.9	Overweight	No	2	0
S0187703418	Male	67	20.99	42.3	Normal	No	0	0
S0187703572	Female	60	27.34	43.8	Overweight	No	0	0
S0187705815	Female	70	20.12	48	Normal	No	2	0
S0187706476	Male	66	23.83	41.2	Normal	No	1	0
S0187707775	Female	76	26.31	45.8	Overweight	No	2	0
S0187711113	Male	62	18.61	46.4	Normal	No	0	0
S0187713528	Female	67	21.08	43.3	Normal	No	0	0
S0187716009	Male	52	23.66	44.6	Normal	No	1	0

**Table 2:** Clinical data of enrolled patients.

Serial number	Site of onset	Degree of differentiation	TNM staging	Completion of chemotherapy	Special drugs were used during the perioperative period
RAG34810	Rectum	Moderately differentiated	T4N0M0	NO	NO
S01669032	Rectum	Moderately to poorly differentiated	T3N1M0	NO	NO
S0187673746	Rectum	Moderately differentiated	T4N0M0	NO	NO
S0187686289	Part of ileocecal junction	Moderately differentiated	T3N1M0	NO	NO
S0187692156	Ascending colon	Moderately differentiated	T3N1M0	NO	NO
S0187693323	Rectum	Moderately differentiated	T2N1M0	NO	NO
S0187693536	Ascending colon	Moderately differentiated	T3N0M0	NO	NO
S0187698716	Descending colon	Moderately differentiated	T4N0M0	NO	NO
S0187698873	Rectum	Moderately to poorly differentiated	T2N1M0	NO	NO
S0187698985	Part of ileocecal junction	Moderately differentiated	T3N1M0	NO	NO
S0187699051	Sigmoid colon	Moderately to poorly differentiated	T3N0M0	NO	NO
S0187701736	Rectum	Moderately to poorly differentiated	T4N1M0	NO	NO
S0187703418	Ascending colon	Moderately differentiated	T4N0M0	NO	NO
S0187703572	Ascending colon	Poorly differentiated	T4N0M0	NO	NO
S0187705815	Sigmoid colon	Moderately differentiated	T3N0M0	NO	NO
S0187706476	Rectum	Moderately to poorly differentiated	T4N0M0	NO	NO
S0187707775	Rectum	Moderately differentiated	T4N0M0	NO	NO
S0187711113	Descending colon	Moderately differentiated	T4N2M0	NO	NO
S0187713528	Transverse colon	Moderately differentiated	T4N0M0	The 4th and 6th cycles of chemotherapy was not performed because of severe gastrointestinal reactions	NO
S0187716009	Rectum	Moderately differentiated	T4N1M0	NO	NO

Inclusion Criteria: (1) Patients aged 50–80 years old. (2) Radical resection of CRC was performed in our hospital and the pathological diagnosis was colorectal adenocarcinoma. (3) Postoperative adjuvant chemotherapy was performed according to the staging. (4) Informed about the research content of the subject and volunteered to cooperate with the research. Exclusion criteria: (1) Probiotics have been used within 1 month prior to specimen collection. (2) During chemotherapy, targeted therapy or radiotherapy may be required due to changes in the condition. (3) Pathological judgment of digestive system tumors that are not research projects, including but not limited to benign and malignant intestinal hyperplasia, such as sarcomas, hemangiomas, neurosecretory tumors and melanoma in the gastrointestinal tract, etc. (4) Individuals who have suffered from any malignant tumors of the digestive system in history or have undergone related surgeries. (5) Patients with adenomatous polyps, hereditary colorectal cancer or Peutz-Jeghers syndrome that have a family genetic predisposition. (6) Patients with inflammatory bowel disease or tuberculosis infection in the intestines. (7) Severe cases suffering from metabolic or infectious diseases including but not limited to refractory hypertension, diabetes, chronic liver disease, etc. (8) Patients who have undergone non-radical surgery. (9) Patients with renal insufficiency before chemotherapy (with blood creatinine values higher than the upper limit of normal). (10)Temporary or permanent stoma was performed.

This study has secured approval from the Ethics Committee of the Second People's Hospital of Three Gorges University (The ethical clearance number: 202439). During stool sample collection, we strictly uphold the principle of voluntariness, making sure that patients are fully informed about the research and voluntarily consent to participate.

### 2.2 Sample processing

For sample processing, fresh fecal specimens are gathered 24 h prior to the first chemotherapy session and 24 h after each chemotherapy cycle, with a minimum volume of 0.5 grams per sample. These samples are placed in sterile, enzyme-free containers and promptly stored in a −20 °C freezer for short-term preservation. Within 24 h, they are transferred to a −80 °C freezer for long-term storage until subsequent 16S rRNA sequencing analysis.

### 2.3 Total DNA extraction and 16S rRNA sequencing

DNA extraction was carried out utilizing the HiPure Stool DNA Mini Kit (Shanghai Maigen Biotechnology Co, Ltd). Following extraction, the DNA concentration was determined using a Qubit instrument, and the integrity of the genomic DNA was evaluated through 1.5% agarose gel electrophoresis. PCR amplification was conducted on a PCR machine with specific primers (341F5′-CCTACGGGNGGCWGCAG3′) and 805R (5′-GACTACHVGGGTATCTAATCC-3′). The PCR reaction mixture had a final volume of 30 μl, comprising 15 μl of 2 × KAPA HiFi, 1 μl each of forward and reverse primers, 12.5 ng of template DNA, and double-distilled water (ddH2O) to reach the desired volume. The PCR protocol involved an initial denaturation step at 95°C for 3 min, followed by 25 cycles of denaturation at 95°C for 30 s, annealing at 55°C for 30 s, and extension at 72°C for 15 s, with a final extension at 72°C for 5 min and storage at 4°C. After PCR, the products were purified using magnetic bead technology, followed by concentration measurement with Qubit, and verification of the PCR product size through 1.5% agarose gel electrophoresis. Subsequently, an amplicon library was constructed by combining the PCR products, followed by the addition of sequencing adapters and another round of PCR amplification to remove primer dimers or small fragments using magnetic beads, while recording the indexing information for each library. The libraries were then pooled and sequenced on platforms like Illumina Miseq or Novaseq, employing a paired-end sequencing mode with 250 or 300 base pairs.

### 2.4 Microbiota analysis

In this study, we employed the “dad2” package in R for data denoising. After eliminating erroneous sequences, we clustered sequences with 100% identity, defining them as ASV (Amplicon Sequence Variant). Subsequently, we normalized the feature table using the “Rarefy” method and based subsequent analyses on this normalized table. For taxonomic annotation, we sourced 16S rRNA sequences from the SILVA database (version 138, SSURef_NR99), filtered the specified regions to remove duplicates, and retained only broadly supported taxonomic information. We then utilized the “fit-classifier-naive-bayes” tool within the “feature-classifier” plugin of QIIME 2 to train a classifier on this database. Finally, the “classify-sklearn” method in the same plugin was applied to annotate the feature sequences. For visualization purposes, we used the “pheatmap” package in R. To evaluate microbial richness and diversity within samples, we calculated alpha diversity indices, including Chao1 (richness estimator), Shannon (diversity index), and Simpson (diversity index), which were computed using the vegan package in R based on the rarefied ASV table, while beta diversity was assessed to compare differences between samples. We used the “ggplo2” package in R to generate PCoA (Principal Coordinate Analysis) plots, visually illustrating sample similarities. To identify statistically significant biomarkers between groups, we conducted LEfSe analysis (Linear Discriminant Analysis Effect Size). Additionally, we predicted the functional abundance of the microbial community using PICRUSt2 (Phylogenetic Investigation of Communities by Reconstruction of Unobserved States), providing deeper insights into the potential functional attributes of the microbial community. Functional predictions were further annotated against the Kyoto Encyclopedia of Genes and Genomes (KEGG) database, and pathway-level abundances were calculated by summing the relative abundances of KEGG Orthologs (KOs) assigned to each pathway, allowing us to evaluate potential associations between microbial functional shifts and chemotherapy-related side effects. The raw sequencing reads generated in this study has been deposited in Sequence Read Archive (SRA) of NCBI, Bioproject accession number: PRJNA1314062.

### 2.5 Statistical analysis

Paired-end sequencing was performed using the Illumina Novaseq platform in this project. After initial data acquisition, rigorous quality control and sequence screening were conducted. Subsequently, during the clustering of Operational Taxonomic Units (OTUs)/Amplicon Sequence Variants (ASVs) and species identification, quantitative analysis at the OTU/ASV level was performed using QIIME software. We assessed the normality of α-diversity indices using Shapiro-Wilk test. Shannon index was normally distributed (*p* > 0.05) and analyzed by *t*-test; Chao1 and Simpson indices deviated from normality (*p* < 0.05) and were analyzed by Mann-Whitney U test. While beta diversity indices were analyzed through permutational multivariate analysis of variance (PERMANOVA) to assess inter-sample differences. LEfSe analysis was applied to determine the effect sizes of significantly different bacterial species, with an LDA Score threshold set at 3. Statistical analyses were conducted using SPSS 21.0 software, and bivariate correlations were assessed using the Spearman test, where a *P*-value of less than 0.05 was deemed statistically significant throughout the analysis. Cohen's *d* was used to calculate the effect size, the formula is *d* = *X*_1_*-X*_2_ */ Sp*. The corresponding 95% confidence interval (CI) of Cohen's *d* was presented to evaluate the reliability of the results. The experimental data were subjected to Benjamini-Hochberg (BH) correction.

## 3 Result

### 3.1 Differences in gut microbiota composition before and after chemotherapy

#### 3.1.1 Alpha diversity

**Figure 1:**
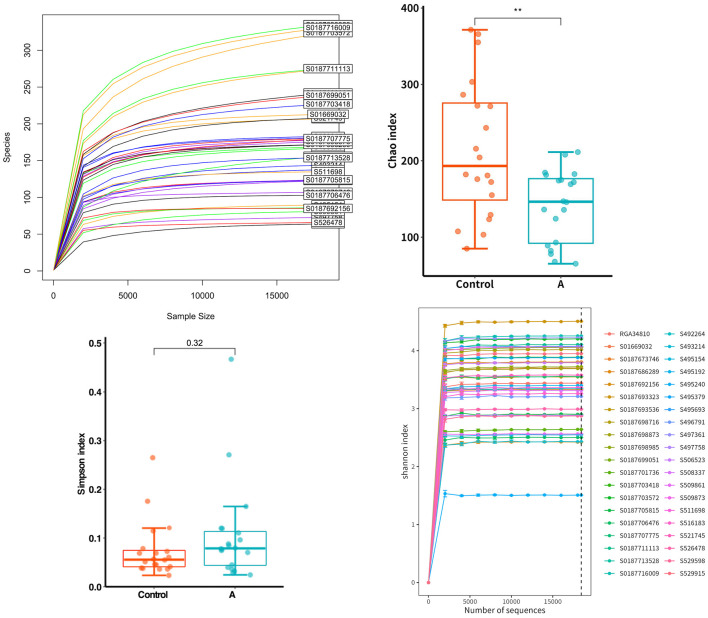
Differences in gut microbiota alpha diversity before and after chemotherapy in the same colorectal cancer patient. A: Rarefaction curve of all patient samples. B: The difference in richness between the pre-chemotherapy baseline group (control) and the post-first chemotherapy group **(A)** was indicated by the Chao1 index, showing a significant difference in richness between the two groups (**p < 0.01) C: The difference in gut microbial diversity between the pre-chemotherapy baseline group (control group) and the post-first chemotherapy group (Group A) was represented by the Simpson index, and there was no significant difference in diversity between the two groups (*P* = 0.22) D:The overall range of the Shannon index (yellow-green curve) before chemotherapy and the Shannon index (red-blue curve) after chemotherapy showed no significant changes in the patient.

In microbiology, alpha diversity serves as a key metric for evaluating species richness and diversity within individual samples. The richness index quantifies the number of distinct biological categories present, while diversity assessments focus on community differences (Kim et al., 2020). In this study, we analyzed the alpha diversity of gut microbiota in the same CRC patients before and after chemotherapy. To begin, we randomly subsampled sequences from each specimen and constructed a Rarefaction Curve (RC) by plotting the number of sequences against the corresponding species count. The observed stabilization of the RC indicated that the sequencing depth was adequate to represent the species diversity within the samples (Figure 1A). Analysis of the Chao1 index revealed a significant reduction in gut microbial community richness in CRC patients post-chemotherapy compared to pre-chemotherapy levels (*P* < 0.01, 95%CI: 0.008–0.011, effect size = 0.405, Figure 1B). Conversely, Simpson index analysis showed no substantial difference in microbial diversity before and after treatment (*P* = 0.32, 95%CI: 0.310–0.329, effect size = 0.16, Figure 1C). Similarly, comparison of the Shannon index—represented by yellow-green (pre-chemotherapy) and red-blue (post-chemotherapy) curves—demonstrated no significant overall changes in gut microbial diversity between the two groups (Figure 1D). These findings suggest that while microbial abundance decreased following chemotherapy, overall community diversity remained largely unchanged, which implied that, despite reductions in certain microbial populations, the gut microbiota's structural and functional integrity was preserved to some extent, highlighting the resilience and stability of the gut microbial ecosystem in CRC patients after chemotherapy.

#### 3.1.2 Classification and cluster analysis of OUT/ASV

**Figure 2:**
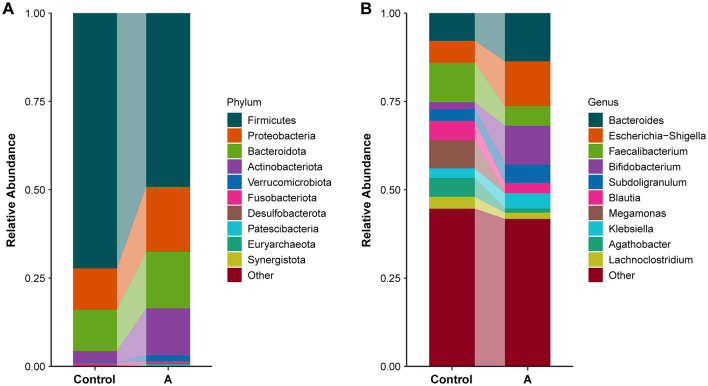
Composition of the gut microbiota. Comparison of the average abundance of bacterial phyla in each group before and after chemotherapy **(A)**. Comparison of the average abundance of bacterial genus in each group before and after chemotherapy **(B)**.

OTUs represent a method for clustering microorganisms based on DNA sequence similarity, with organisms sharing at least 97% similarity grouped into the same OTU. This approach enables the depiction of microbial community composition across various taxonomic ranks, including kingdom, phylum, class, order, family, genus, and species. When erroneous sequences are excluded and the clustering threshold is set at 100% similarity, the resulting units are termed Amplicon Sequencing Variants (ASVs; Schloss and Handelsman, 2005). In analyzing the microbial community composition before and after chemotherapy, the top 10 most abundant phyla and genera are illustrated in the pictures. At the phylum level, both sample groups were primarily composed of *Firmicutes, Proteobacteria, Bacteroidetes, Actinobacteria, and Verrucomicrobia*. Post-chemotherapy, there was an increase in the relative abundance of *Proteobacteria, Actinobacteria, and Verrucomicrobia*, whereas *Firmicutes* and *Bacteroidetes* showed a significant decrease (Figure 2A). At the genus level, the dominant genera in both sample groups included *Bacteroides, Escherichia-Shigella, Faecalibacterium, Bifidobacterium, Subdoligranulum, Blautia, Megamonas, Klebsiella, Anaerostipes, and Clostridium*. After chemotherapy, the abundance of B*acteroides, Escherichia-Shigella, Bifidobacterium, Subdoligranulum, and Klebsiella* increased significantly compared to pre-chemotherapy levels, while Faecalibacterium, Blautia, Megamonas, Anaerostipes, and Clostridium experienced a notable decline (Figure 2B).

#### 3.1.3 Beta diversity

**Figure 3:**
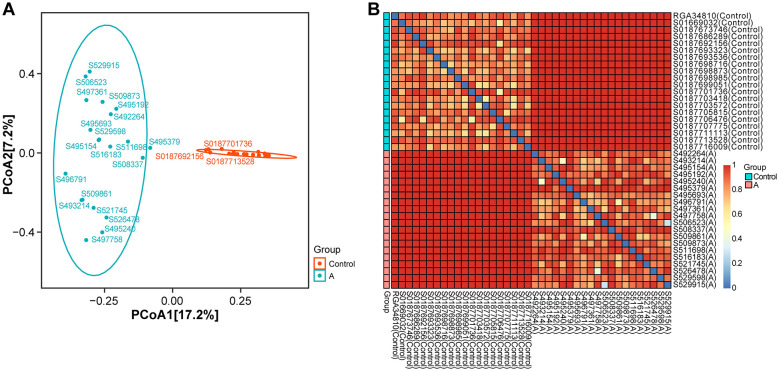
The distribution of gut microbiota across all samples. PCoA was applied to demonstrate the distribution of gut microbial communities among the samples, each symbol represents a sample (red, pre-chemotherapy control group; blue, post-chemotherapy group A) **(A)**. Significant difference species distance heatmap (Bray–Curtis's distance metric of the 20 samples) **(B)**.

Beta diversity analysis evaluates the dissimilarity in microbial community composition between two groups—in this case, samples collected before and after chemotherapy. A smaller beta diversity value indicates greater similarity in species composition between the groups. Principal Coordinate Analysis (PCoA) was performed at the genus level to depict the distribution of microbial communities across samples (Figure 3A). The first principal component (x-axis) accounted for 17.2% of the total data variation, while the second principal component (y-axis) explained 7.2%. The PCoA plot revealed a clear separation in gut microbial composition between pre- and post-chemotherapy groups, indicating significant shifts in community structure. Additionally, a heatmap illustrating the distances between significantly differential species confirmed marked differences in abundance patterns between the two groups (Figure 3B).

#### 3.1.4 LEfSe analysis

**Figure 4:**
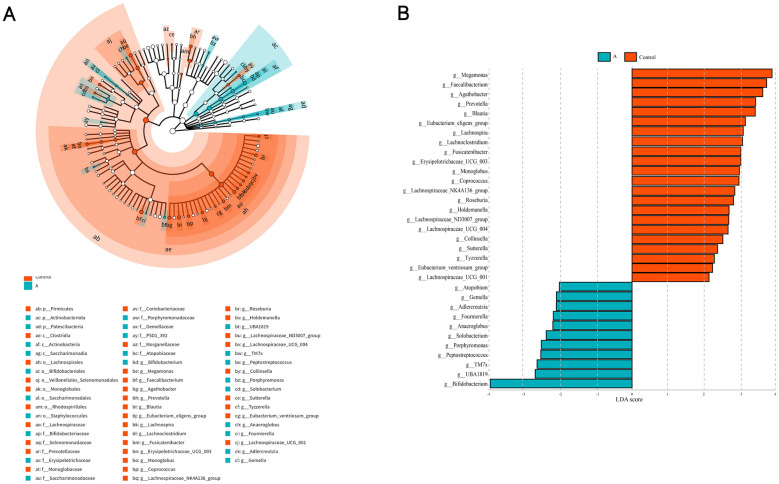
Gut microbiota associated with chemotherapy. The phylogenetic tree illustrates the differences in gut microbial abundance between the pre-chemotherapy and post-chemotherapy groups **(A)** Linear discriminant analysis (LDA) effect size (LEfSe) analysis of changes in gut microbiota after chemotherapy.Linear discriminant analysis of differentially abundant genera indicates their contribution to group differentiation. The cladogram using the LEfSe method displays the phylogenetic distribution of gut microbiota in patients before (red) and after (blue) chemotherapy, with an LDA score threshold set at 3 **(B)**.

LEfSe analysis was utilized to discern key differences in gut microbiota composition between pre- and post-chemotherapy samples, employing an LDA score threshold of ≥ 3 to identify statistically significant bacterial taxa. The pre-chemotherapy gut microbiota was primarily characterized by *Megamonas, Faecalibacterium, Agathobacter, Prevotella*, and *Blautia*, while the post-chemotherapy group exhibited a notable enrichment of *Bifidobacterium* (Figure 4A and B).

### 3.2 Analysis of gut microbial differences across different chemotherapy cycles

**Figure 5:**
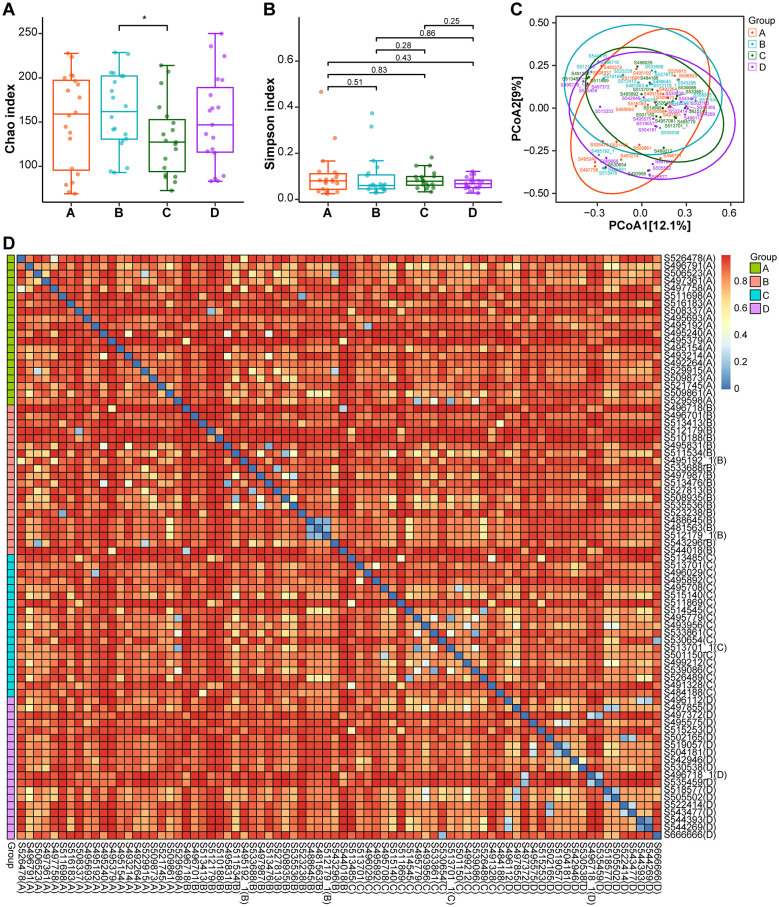
Analysis of gut microbial differences across different chemotherapy cycles (GroupA: after the 1st cycle, GroupB: after the 3rd cycle, GroupC: after the 4th cycle, GroupD: after the 6th cycle) **(A)** Chao1 index, comparison of gut microbial richness differences among the four groups **(B)** Simpson index, compare the differences in gut microbial diversity among the four groups **(C)** PCoA, principal coordinate analysis Plot of gut microbiota among the four groups **(D)** Significant difference species distance heatmap among four groups.

The results of the above studies showed that the composition and structure of the gut microbiota changed significantly after chemotherapy compared with that before chemotherapy, suggesting that chemotherapy may be a related factor affecting the composition of gut microbiota. Given that chemotherapy usually involves multiple cycles rather than a single treatment, in order to further evaluate the relationship between chemotherapy and microbiota, we further analyzed microbial communities in different chemotherapy cycles to assess the longitudinal stability of microbial community structure under cumulative chemotherapy and explore the cumulative effects of successive chemotherapy cycles on the dynamics of gut microbiota. To explore this, we conducted the same tests on fecal samples from these 20 patients at different chemotherapy cycles (GroupA: after the 1st cycle, GroupB: after the 3rd cycle, GroupC: after the 4th cycle, GroupD: after the 6th cycle), and found that significant differences in microbial richness were only observed between Group B and Group C (*P* < 0.05, 95%CI: 0.321–0.339, effect size = 0.11, Figure 5A), while no differences in richness or diversity were detected among the other groups. In addition, principal coordinates analysis (PCoA) plots and inter-sample distance heatmaps indicated no obvious differences in the overall composition of gut microbiota across all groups (Figure 5C and D). These findings suggest that alterations in gut microbiota composition may not follow regular or dynamic changes along with the progression of chemotherapy cycles. But considering the relatively small sample size of this study, further in-depth investigations are required in this field in the future.

### 3.3 The relationship between gut microbiota composition and chemotherapy side effects

**Figure 6:**
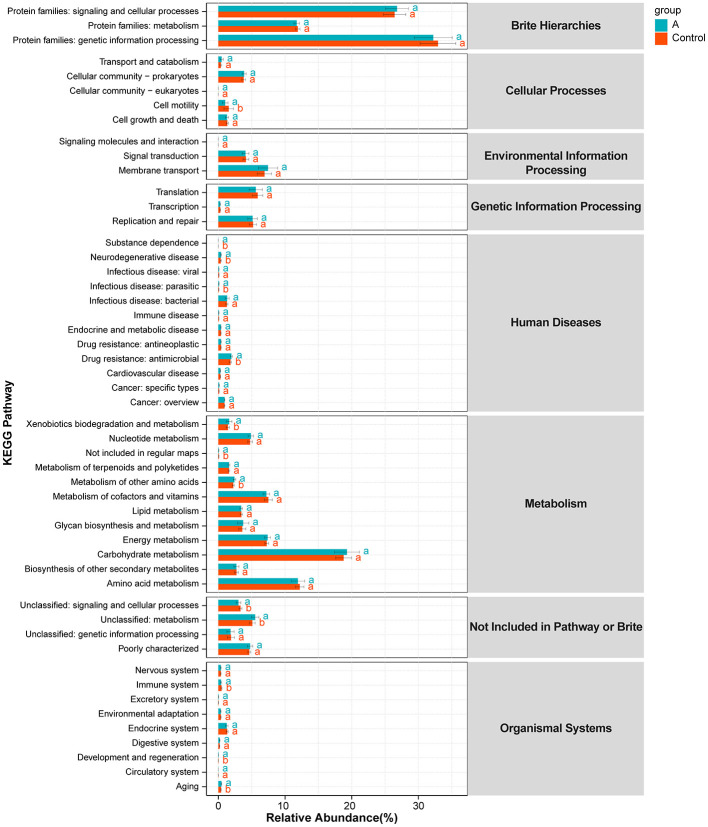
Bar chart of KEGG functional relative abundance in pre-chemotherapy and post-chemotherapy groups.

Chemotherapy side effects represent a significant clinical concern, as gut microbiota play a pivotal role in modulating physiological processes, including nervous system function, gut function, and the immune system (Feng et al., 2022). We hypothesized that cumulative chemotherapy cycles may induce alterations in gut microbiota composition, which in turn could contribute to chemotherapy side effects through dysregulation of relevant genes or metabolic pathways. To investigate this, we predicted the relative abundance of KOs unctional genes using 16S rRNA gene sequencing data, and pathway-level abundances were calculated by aggregating the relative abundances of KOs associated with each pathway, enabling exploration of the potential relationship between gut microbiota and chemotherapy side effects. From the analysis results, pathways related to metabolism, genetic information processing, and bacteria were significantly upregulated after chemotherapy, while pathways related to cell motility, nervous system function, and the immune system were markedly downregulated after chemotherapy (Figure 6). These data suggest that chemotherapeutic agents substantially alter gut microbiota composition, with microbial shifts potentially influencing immune function and nutritional status of CRC patients. Such changes may thereby contribute to the development of chemotherapy-related adverse effects, highlighting the interplay between microbial dynamics and treatment outcomes.

## 4 Discussion

CRC remains a major global health challenge due to its high incidence and mortality rates. While surgical resection serves as the definitive treatment for colon cancer, postoperative adjuvant chemotherapy plays a critical role in eliminating micro-metastases and improving long-term survival outcomes (Chan and Chee, 2019). Emerging evidence indicates that alterations in gut microbiota composition significantly inflience CRC development and progression (Li et al., 2025). Certain pathogenic species, including *Bacteroides fragilis* and specific *Escherichia coli* strains, contribute to gut damage through toxin production and exhibit positive correlations with CRC incidence (Anna et al., 2024; Christine et al., 2018). Similarly, *Fusobacterium nucleatum* demonstrates increased abundance in adenomatous tissues, with levels escalating alongside disease progression. This bacterium not only persists within CRC epithelial cells but also shows potential as a predictive biomarker for chemotherapy response (Shinichi et al., 2019). Conversely, probiotic-derived short-chain fatty acids like butyrate, produced through dietary polysaccharide fermentation, have demonstrated antitumor properties by inhibiting tumor growth (Li et al., 2024; Zhao et al., 2023). Despite these insights, studies examining the relationship between chemotherapy and gut microbiota dynamics in CRC patients remain limited. The 16S rRNA gene sequencing approach has emerged as an effective tool for comprehensive microbial community analysis (Caporaso et al., 2011). In this study, the 16S rRNA sequencing method was employed to investigate the differences in gut microbiota of the same CRC patient before chemotherapy and after different cycles of chemotherapy. The changes in the composition of gut microbiota before and after chemotherapy were analyzed, and a preliminary correlation between the experimental results and the adverse reactions following CRC chemotherapy was hypothesized.

This study employed the Chao1 index to assess microbial richness and the Shannon and Simpson indices to evaluate diversity, analyzing gut microbiota composition in CRC patients before and after chemotherapy. The results demonstrated a significant reduction in microbial richness post-treatment compared to baseline, while overall diversity remained statistically unchanged. This pattern suggests that although chemotherapy selectively depleted certain microbial taxa, the community retained its fundamental structural and functional integrity, may indicating inherent resilience and stability in the post-treatment gut microbiota. It is evident that chemotherapeutic agents may effect microbial populations implicated in CRC progression or treatment resistance, thereby contributing to therapeutic efficacy through microbiota modulation. The observed stability in microbial diversity despite reduced richness implies that targeted microbial interventions may have the potential to improve treatment outcomes. This finding offers a reference for future research on microbiota-related therapeutic strategies for CRC, which requires further verification in clinical studies.

In addition, we also observed significant changes in the major components of the gut microbiota between the two groups before and after chemotherapy. At the phylum level, post-treatment samples exhibited reduced proportions of *Firmicutes and Bacteroidetes*, with concurrent increases in *Proteobacteria, Actinobacteria*, and *Verrucomicrobia*. At the genus level, *Bacteroides, Escherichia-Shigella, and Bifidobacterium* showed elevated abundances, while *Faecalibacterium and Blautia* decreased. These findings align with prior research demonstrating Firmicutes and Bacteroidetes dominated pre-treatment microbial communities in CRC populations, chemotherapy-induced reductions in microbial diversity, *Firmicutes* abundance, and *Faecalibacterium* levels, alongside increases in *Proteobacteria and Verrucomicrobia* (Huang et al., 2023; Zhao et al., 2024). Our results revealed a decline in the abundance of pathogenic Bacteroides after chemotherapy. Existing studies have confirmed that Bacteroides fragilis can secrete toxins to activate the TLR4-NFAT5 pathway and upregulate JMJD2B, thereby promoting the stemness of CRC cells (Liu et al., 2020). Combined with our observations, this genus may be potentially associated with chemotherapy response in CRC patients. Conversely, the abundance of Bifidobacterium increased following oxaliplatin-based chemotherapy. Previous studies have indicated that probiotics such as Bifidobacterium, Lactobacillus acidophilus, and Streptococcus can potentially enhance intestinal barrier function and maintain gut homeostasis *via* multiple pathways including short-chain fatty acid synthesis, immune regulation and pathogen inhibition (Bao et al., 2022; Lu et al., 2019). The upregulation of Bifidobacterium in our study may serve as a potential indicator of improved gut microecology. These results suggest that the gut microbiota composition appears healthier following chemotherapy. However, it is noteworthy that LEfSe analysis also indicated significant enrichment of *Blautia* prior to chemotherapy. *Blautia*, an important anaerobic gut bacterium, produces butyrate through dietary fiber fermentation, exerting anti-inflammatory and anti-tumor effects (Liu et al., 2021). Together with *Faecalibacterium*, it constitutes a key butyrate-producing bacterial group. Its significant reduction after chemotherapy suggests that drug toxicity may compromise intestinal barrier integrity and exacerbate inflammatory responses. These findings raise the question of whether a dynamic equilibrium exists between chemotherapeutic agents and the gut microbiota. Future research and clinical translation should therefore focus on optimizing chemotherapeutic dosing regimens or consider targeted supplementation of specific butyrate-producing bacteria during chemotherapy to re-establish and maintain a dynamic balance between therapeutic efficacy and intestinal microecological homeostasis, thereby improving patient treatment tolerance and long-term outcomes.

Emerging evidence indicates that the interaction between the gut microbiota and chemotherapy drugs such as 5-fluorouracil (5-FU), irinotecan, and oxaliplatin is extremely complex. The microbiota not only passively responds to chemotherapy but also actively metabolizes the chemotherapy drugs through specific enzyme systems, directly regulating the efficacy and toxicity of the drugs (Despoina et al., 2023; Wang and Zhou, 2025). Iida et al. (2013). demonstrated that germ-free or antibiotic-treated mice exhibited significantly diminished therapeutic responses to oxaliplatin. The underlying mechanism involves gut commensal bacteria modulating oxaliplatin's antitumor efficacy through the regulation of ROS production and innate immune responses. A 2017 studyfurther revealed that *Gram-positive bacteria*, including *Lactobacillus* and *Enterococcus species*, potentiate oxaliplatin-induced DNA damage and apoptosis *via* peptidoglycan-mediated innate immune signaling pathways (Alexander et al., 2017). More recently, Fang et al. (2025) identified that gut microbiota-derived deoxycholic acid enhances FOLFOX therapeutic efficacy through Ugt1a6b-mediated enterohepatic circulation in colorectal cancer. Collectively, these findings illustrate a bidirectional relationship: chemotherapy reshapes microbiota composition, while microbial alterations reciprocally influence chemotherapeutic outcomes. Previous investigations have established that elevated abundance of *Bacteroides* fragilis correlates with chemotherapy resistance and unfavorable prognosis in colorectal cancer (Ding et al., 2025). Modulating *Bacteroidetes* levels may represent a viable strategy to overcome resistance mechanisms and improve therapeutic efficacy through microbiota intervention. However, further in-depth research is warranted to elucidate the precise roles of specific microbial communities and their metabolites in CRC pathogenesis, as well as the molecular mechanisms underlying chemotherapy-induced microbial shifts, and such investigations could inform targeted microbiota interventions to optimize therapeutic. Relevant findings may provide theoretical references for developing targeted microbiota interventions to improve treatment efficacy and reduce adverse reactions.

While the combination of radical surgery and chemotherapy has significantly improved survival rates in CRC patients, chemotherapy side effects frequently impede treatment completion, with recurrence and distant metastasis remaining critical contributors to mortality. Dysbiosis of the symbiotic gut microbiota has been implicated in systemic disease progression through modulation of host metabolic and immune responses (Mao et al., 2021). Our findings demonstrate significant post-chemotherapy alterations in gut microbial composition, particularly a marked increase in *Proteobacteria* abundance—a recognized biomarker of microbial imbalance (Shin et al., 2015). Such dysbiosis correlates with common chemotherapy-induced gastrointestinal toxicities such as vomiting and diarrhea (Chang et al., 2021; Li et al., 2022), suggesting a potential link between microbiota disruption and treatment side effects. KEGG pathway analysis of 16S rRNA sequencing data revealed post-chemotherapy upregulation of bacterial metabolism and genetic information processing pathways, while immune system, nervous system, and cell motility pathways were downregulated. Myelosuppression represents a prevalent and clinically significant toxic side effect of chemotherapy, often manifesting as leukopenia or neutropenia (Zhao et al., 2020). This reduction in white blood cell counts heightens patients' vulnerability to bacterial infections, which can subsequently trigger systemic inflammatory responses (Ni et al., 2023; Sharma et al., 2024). Such chemotherapy-induced systemic inflammation critically influences both treatment tolerance and long-term prognosis in CRC patients, with inflammatory processes playing pivotal roles in postoperative complications, disease progression, impaired immune function, and wound healing (Li et al., 2025; Shu et al., 2025; Wang et al., 2025). Previous studies reported that chemotherapy-related cognitive impairment may be caused by the direct neurotoxicity of chemotherapeutic drugs (Chen et al., 2024), and the decreased abundance of Bacteroides after chemotherapy is also speculated to be a potential influencing factor (Otto-Dobos et al., 2024). Our study confirmed the reduction of Bacteroides after treatment, but the causal relationship between this genus and cognitive decline remains unclear. Concurrently, our 16SrRNA sequencing data revealed increased abundances of *Escherichia coli and Shigella* species following chemotherapy, and these pathogens are known to modify the tumor microenvironment, accelerate CRC progression, and enhance chemoresistance (Garvey, 2024; Nilmara de Oliveira et al., 2024); similarly, certain *Klebsiella* species, which also increased post-treatment, secrete biofilms and virulence factors that compromise immune responses and elevate infection risk (Bai and Guo, 2024; Palusiak, 2022). These bacteria are associated with chemotherapy side effects such as infection, myelosuppression, and hypoglycemia, and their abundance significantly increased after chemotherapy in our 16SrRNA sequencing data. In addition, chemotherapy can also affect the metabolic processes of proteins and carbohydrates, leading to changes in energy production and material synthesis, thereby affecting the patient's energy levels and overall metabolic status (Timia et al., 2020). These metabolic changes contribute to malnutrition, which in turn impairs immune function and delays postoperative wound healing, further exacerbating the occurrence of complications (Shayimu et al., 2024; Zhang et al., 2025). Interestingly, KEGG pathway analysis suggested enhanced overall metabolic activity post-chemotherapy, potentially linked to increased *Bifidobacterium* abundance (Fushinobu and Abou Hachem, 2021). This observation aligns with the known metabolic benefits of certain probiotic species. Collectively, these findings demonstrate the differential impacts of specific microbial taxa on host physiology during chemotherapy, and we hypothesize that cumulative chemotherapy sessions induce progressive microbial dysbiosis, with bacterial metabolites and immune interactions accumulating to produce treatment-related toxicities. However, it is crucial to recognize that functional predictions based on 16S rRNA sequencing represent indirect inferences and may not fully correspond to results obtained through direct metagenomic validation. Future research should employ multi-omics approaches, including metagenomic sequencing, to precisely elucidate the molecular mechanisms linking gut microbiota composition and chemotherapy outcomes. Such studies would provide essential insights for developing microbiota-targeted interventions to optimize CRC treatment efficacy and minimize adverse effects.

For a long time, cancer has been predominantly viewed as a genetic disorder, but an increasing number of research findings have revealed that the microbiome may play a role in promoting tumor formation (Anderson and Sears, 2023), which is particularly evident in CRC research, where fecal microbiome composition has been strongly implicated in cancer development (Osman and Luke, 2019). Chemotherapy, while a cornerstone of cancer treatment, profoundly disrupts microbial homeostasis, particularly in the digestive tract, by altering the natural structure of microorganisms. This imbalance of microorganisms, in turn, can have a negative impact on the effectiveness of chemotherapy and affect the recovery process of patients. As research progresses, the complex interactions between gut microbiota and chemotherapy drugs, as well as their impact on treatment outcomes and patient responses, are becoming increasingly clear (Aafrin et al., 2022). For instance, Chen et al. (2024) demonstrated that Danggui Sini decoction, a traditional Chinese medicine formulation, could restore gut barrier damage, gut microbiota imbalance, and systemic metabolic disorders caused by oxaliplatin, thereby alleviating oxaliplatin-induced neuropathy. Chang et al. (2021) reported that fecal microbiota transplantation from healthy wild-type mice to CRC-bearing mice effectively mitigated severe diarrhea, bacterial translocation, and gut mucosal damage induced by the FOLFOX chemotherapy regimen, ultimately improving long-term survival rates in treated mice. Although the clinical application value of FMT still needs to be verified in human trials. These findings underscore the tremendous potential of microbiome-based strategies to enhance cancer treatment efficacy. However, the field faces significant challenges, with insufficient research on specific molecular mechanisms, making it difficult to clarify the deep connection between the microbiome and cancer; limited animal experimentation and clinical data make it difficult to fully validate the reliability and effectiveness of microbiome characteristics as diagnostic biomarkers. To address these challenges, future research should focus on expanding sample sizes and employing diverse detection technologies to conduct comprehensive gut microbiota analyses, and well-designed clinical intervention trials are essential to verify the feasibility and effectiveness of microbiome-based approaches in cancer treatment. By overcoming these obstacles, we can pave the way for the development of novel, microbiome-targeted therapies that optimize treatment outcomes and improve the quality of life for cancer patients.

But this study still has certain limitations. Firstly, the small sample size may have limited our ability to detect statistically significant differences. In addition, we collected samples from the same patients across different chemotherapy cycles, yielding data with repeated measures characteristics. However, we did not apply a repeated measures model and instead analyzed these samples as independent data. Even though this was a preliminary exploratory study, this analytical approach compromised the overall rigor of the research. In future work, we will perform standardized longitudinal repeated measures analysis and expand the sample size to improve the robustness and precision of the results. Secondly, the absence of a healthy control group in this study restricts our ability to conduct a more nuanced analysis of gut microbiota composition across different populations. A comprehensive comparison among healthy individuals, CRC patients, and post-chemotherapy patients would facilitate the identification of specific bacterial taxa that could serve as potential therapeutic targets. And the dietary factors represent a significant confounding variable in gut microbiota research, as diet has a profound impact on microbial community structure (Armin et al., 2024; Soldán et al., 2024). While this study attempted to standardize the dietary conditions of enrolled patients through preliminary screening, strict dietary control was not implemented during the study period. Furthermore, all participants were recruited from a single prefecture-level city, this sampling strategy failed to adequately account for the potential influence of regional characteristics on the representativeness of the microbiota. Environmental factors varying across geographical regions, such as climatic conditions, dietary habits, water quality and population genetic backgrounds, may substantially shape the composition and functional profiles of the gut microbiome. Consequently, these limitations may constrain the generalizability of our findings to other ethnic groups or geographical populations. Subsequent studies should endeavor to recruit patients from diverse geographical regions and enforce rigorous dietary protocols, including standardized control of dietary intake, antibiotic usage, and probiotic supplementation, and this approach would help minimize the confounding effects of dietary variations and enhance the validity of the results. Finally, in the short term, constrained by the current cohort size and experimental conditions, we are unable to immediately conduct functional validation experiments, such as fecal microbiota transplantation, germ-free mice, or targeted metabolomics. However, in the future, we will further expand the sample size and simultaneously initiate research on the interaction between gut microbiota and miRNAs in colorectal cancer patients, aiming to delve deeper into their molecular mechanisms and clinical significance. This will provide more robust experimental evidence for the associations between specific enriched or depleted bacterial genera and inflammatory, metabolic, or immune pathways, ultimately elucidating the molecular mechanisms by which they mediate the side effects of chemotherapy.

## 5 Conclusion

This exploratory study revealed that gut microbiota composition in patients did not exhibit sustained dynamic changes throughout the entire observation period, with significant microbial structural differences observed only between the baseline and first-cycle post-chemotherapy groups, which may be associated with patients' initial exposure to chemotherapeutic agents. KEGG functional analysis indicated that gut microbial alterations were potentially correlated with a series of chemotherapy-induced adverse events, including gastrointestinal dysfunction, infection, myelosuppression, and peripheral neuropathy. Collectively, these findings suggest that gut microbiota profiling may serve as a valuable auxiliary indicator for evaluating chemotherapy-related adverse reactions. For instance, increased fecal abundance of Bacteroidetes following chemotherapy may imply a higher risk of chemotherapy resistance, whereas altered Proteobacteria abundance is correlated with the occurrence of gastrointestinal adverse reactions. Nevertheless, these preliminary conclusions require further validation in larger sample cohorts. Current research indicates that supplementation with specific probiotics such as Bifidobacterium, as well as fecal microbiota transplantation, can restore intestinal barrier integrity and the secretion of beneficial metabolites, may help alleviating chemotherapy-triggered immune and neurological complications. Based on the preliminary findings of the present study, further exploration of the molecular mechanisms underlying gut microbiome modulation, together with continuous monitoring and targeted intervention of gut microbiota, may facilitate the development of novel adjuvant strategies to optimize chemotherapy outcomes in patients with colorectal cancer.

## Data Availability

The raw sequencing reads generated in this study have been deposited in the NCBI Sequence Read Archive (SRA) (https://www.ncbi.nlm.nih.gov/sra), Bioproject accession number: PRJNA1314062.
